# Supplementary material for: Cell populations in human breast cancers are molecularly and biologically distinct with age
Source: Nat Aging. 2025 Nov 4;5(12):2546–63. doi: 10.1038/s43587-025-00984-1 (PMC12705435; doi:10.1038/s43587-025-00984-1)
Supplement: Supplementary file 1 — Supplementary Figs. 1–10 with legends. [file 43587_2025_984_MOESM1_ESM.pdf]

nature aging

Supplementary information https://doi.org/10.1038/s43587-025-00984-1

Cell populations in human breast cancers are molecularly and biologically distinct with age

In the format provided by the authors and unedited

TNBC **Supplementary Figure 1**

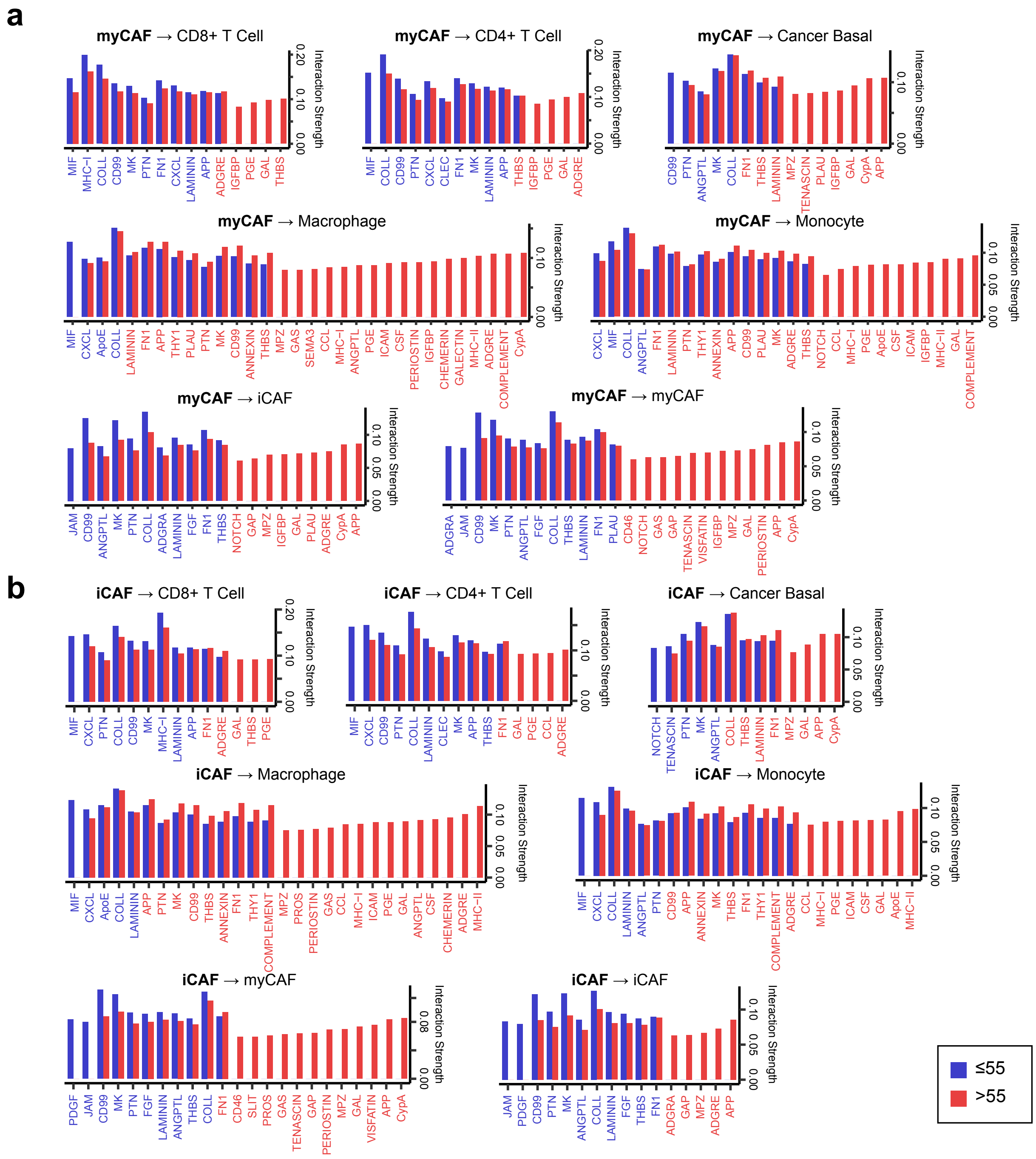

Supplementary Figure 1 | Age-related predicted signaling pathways between highly interactive cell types in TNBC, CAFs (related to Fig. 6). Seven cell types with the highest differential interaction strengths were analyzed to identify specific biological interactions through which they were communicating using the CellChat *rankNet* function. **a-b**, Bar charts depict predicted signaling pathway interaction strength between a given source cell and each of the seven other selected cell types as targets and are organized by source cell type: myCAF (**a**), iCAF (**b**). Red bars indicate interactions in the >55 cohort; blue bars indicate interactions in the ≤55 cohort; text color indicates statistically significant enrichment via Wilcoxon rank-sum test (p-value < 0.05) using permutation-based CellChat communication probability calculations[30] in the >55 group (red), the ≤55 group (blue), or failure to achieve statistical significance (black). Y-axis depicts the scaled interaction probability.

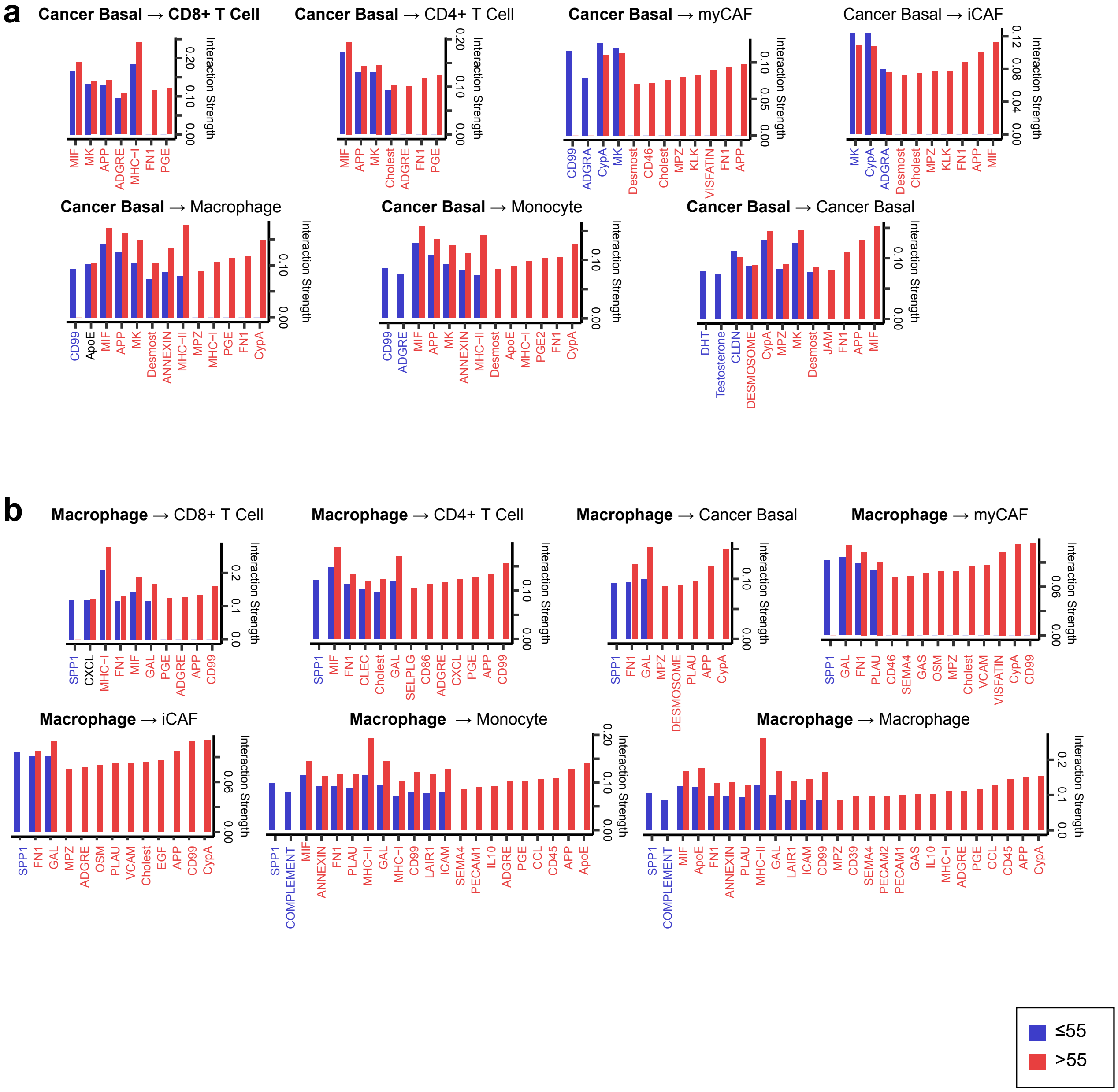

Supplementary Figure 2 | Age-related predicted signaling pathways between highly interactive cell types in TNBC, Basal Cancer Epithelium and Macrophages (related to Fig. 6). Seven cell types with the highest differential interaction strengths were analyzed to identify specific biological interactions through which they were communicating using the CellChat *rankNet* function. **a-b**, Bar charts depict predicted signaling pathway interaction strength between a given source cell and each of the seven other selected cell types as targets and are organized by source cell type: Cancer basal (**a**), Macrophage (**b**). Red bars indicate interactions in the >55 cohort; blue bars indicate interactions in the ≤55 cohort; text color indicates statistically significant enrichment via Wilcoxon rank-sum test (p-value < 0.05) using permutation-based CellChat communication probability calculations[30] in the >55 group (red), the ≤55 group (blue), or failure to achieve statistical significance (black). Y-axis depicts the scaled interaction probability.

Supplementary Figure 3

TNBC

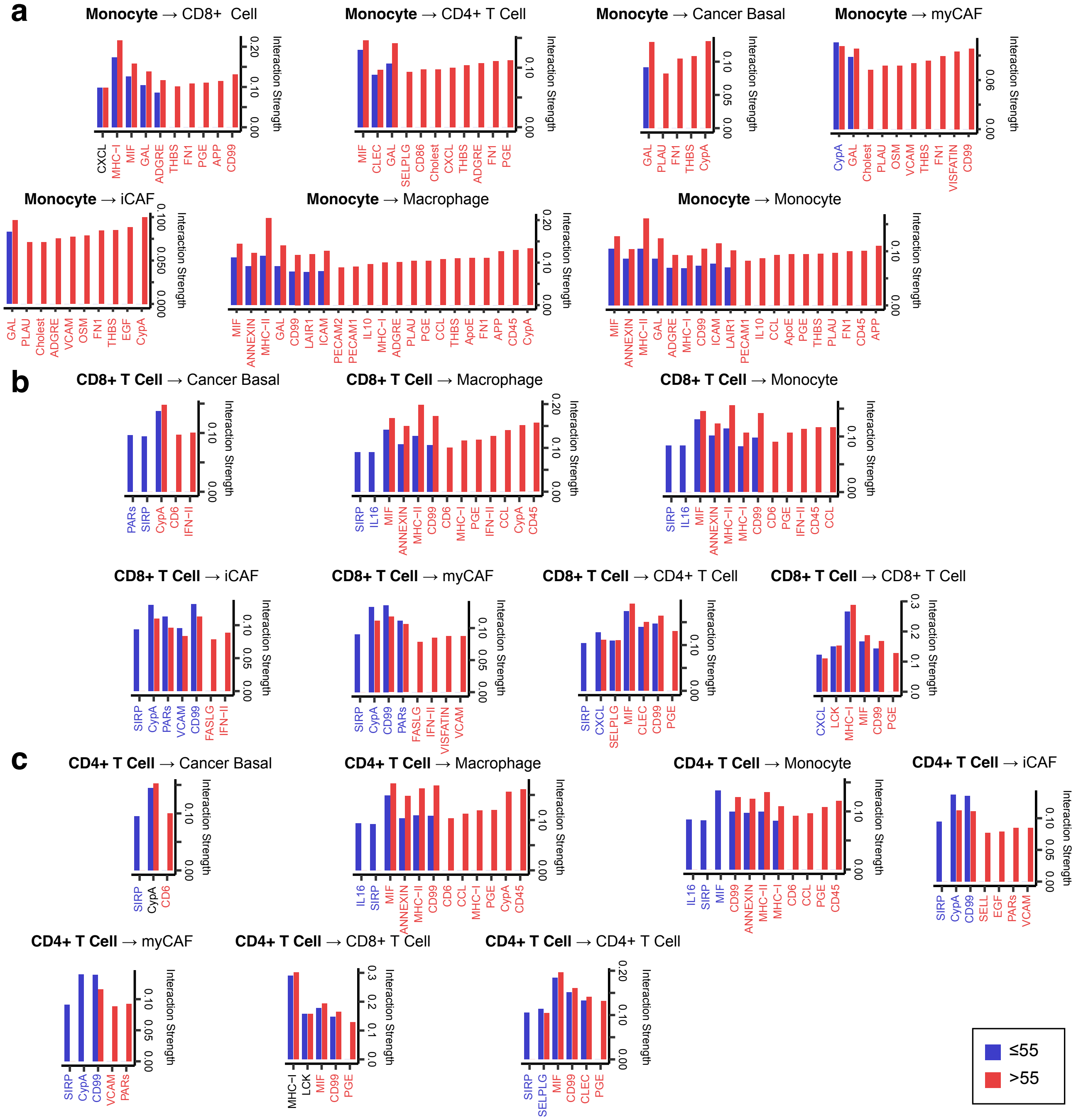

Supplementary Figure 3 | Age-related predicted signaling pathways between highly interactive cell types in TNBC, Monocytes and T Cells (related to Fig. 6). Seven cell types with the highest differential interaction strengths were analyzed to identify specific biological interactions through which they were communicating using the CellChat *rankNet* function. **a-c**, Bar charts depict predicted signaling pathway interaction strength between a given source cell and each of the seven other selected cell types as targets and are organized by source cell type: Monocyte (**a**), CD8+ T Cell (**b**), CD4+ T Cell (**c**). Red bars indicate interactions in the >55 cohort; blue bars indicate interactions in the ≤55 cohort; text color indicates statistically significant enrichment via Wilcoxon rank-sum test (p-value < 0.05) using permutation-based CellChat communication probability calculations[30] in the >55 group (red), the ≤55 group (blue), or failure to achieve statistical significance (black). Y-axis depicts the scaled interaction probability.

Supplementary Figure 4

TNBC

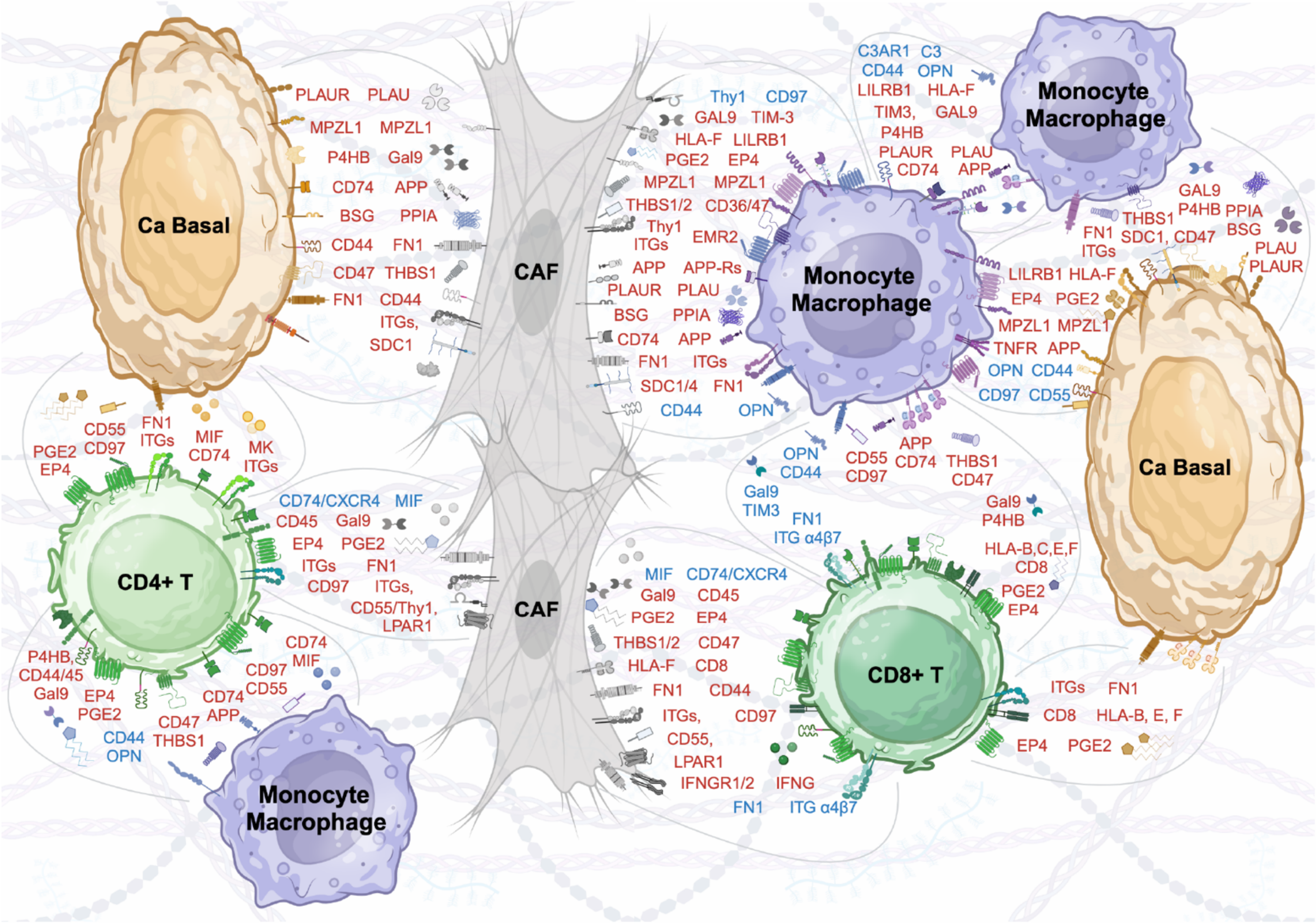

Supplementary Figure 4 | Age-associated signaling nodes in TNBC (related to Fig. 6). Schematic representation of the signaling nodes in TNBC and additional signaling nodes of interest following manual curation of specific cell-cell interactions (See Methods, Source Data Table 5, and Supplementary Figs. 1-3). For clarity of representation, data were combined for iCAFs/myCAFs and monocytes and macrophages Blue text indicates enrichment in the ≤55 age group; red text indicates enrichment in the >55 age group. Created in BioRender. McAllister, S. (2025) . https://BioRender.com/9kvzmpx.

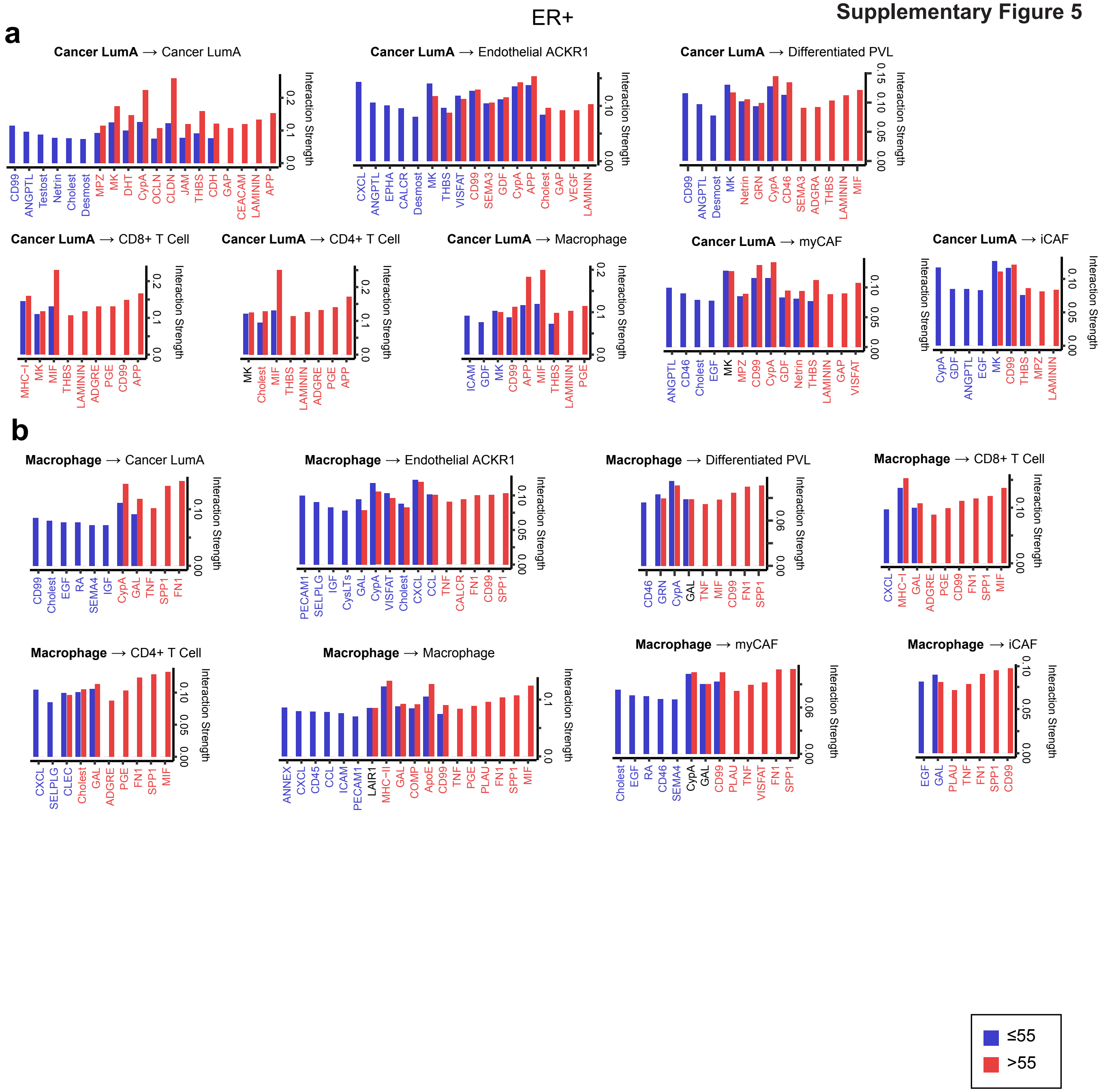

Supplementary Figure 5 | Age-related predicted signaling pathways between highly interactive cell types in ER+ Breast Cancer, Cancer Epithelium and Macrophages (related to Fig. 6). Eight cell types with the highest differential interaction strengths were analyzed to identify specific biological interactions through which they were communicating using the CellChat *rankNet* function. **a-b**, Bar charts depict predicted signaling pathway interaction strength between a given source cell and each of the seven other selected cell types as targets and are organized by source cell type: myCAF (**a**), iCAF (**b**). Red bars indicate interactions in the >55 cohort; blue bars indicate interactions in the ≤55 cohort; text color indicates statistically significant enrichment via Wilcoxon rank-sum test (p-value < 0.05) using permutation-based CellChat communication probability calculations[30] in the >55 group (red), the ≤55 group (blue), or failure to achieve statistical significance (black). Y-axis depicts the scaled interaction probability.

ER+

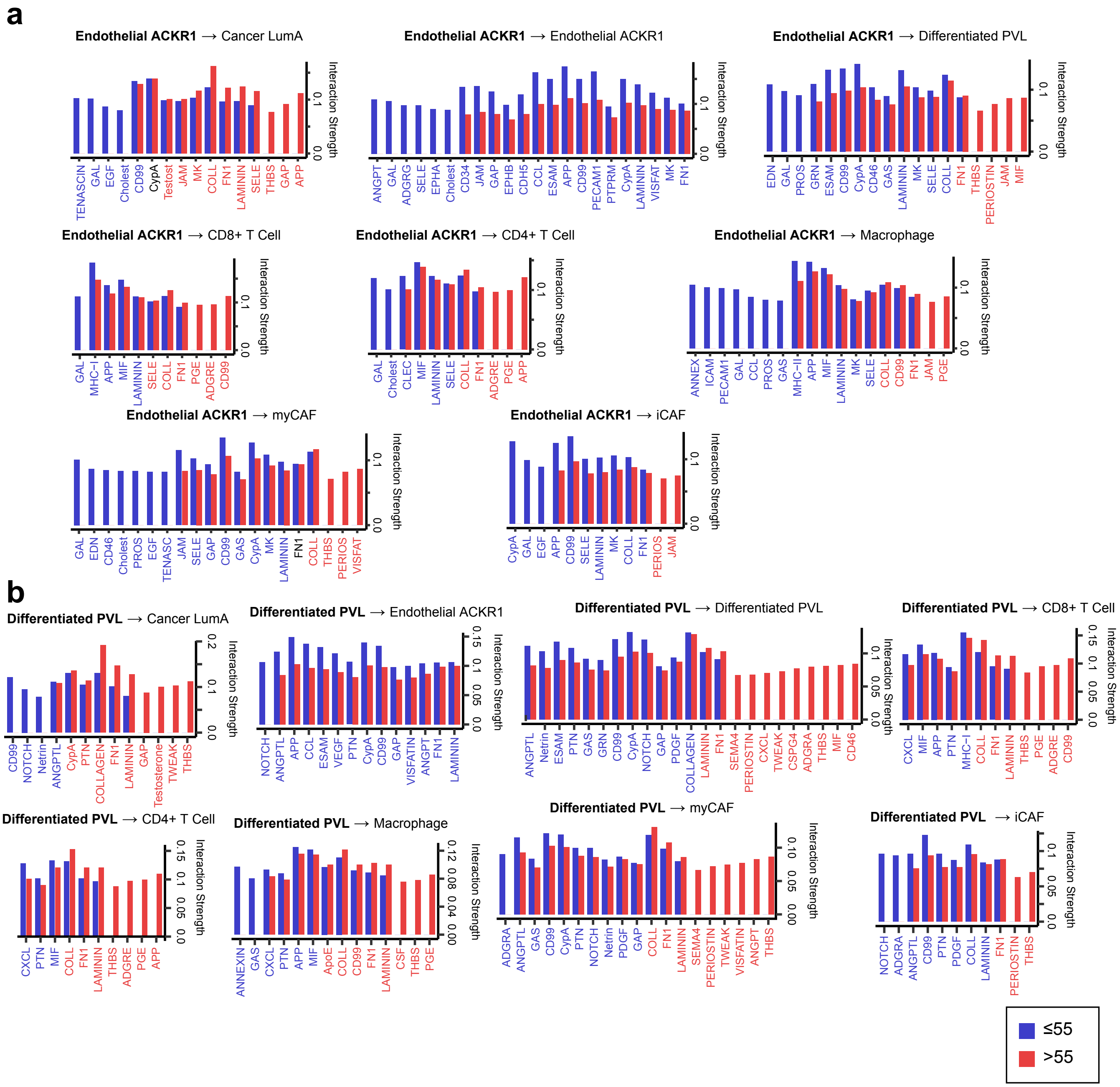

Supplementary Figure 6 | Age-related predicted signaling pathways between highly interactive cell types in ER+ Breast Cancer, Vasculature (related to Fig. 6). Eight cell types with the highest differential interaction strengths were analyzed to identify specific biological interactions through which they were communicating using the CellChat *rankNet* function. **a-b**, Bar charts depict predicted signaling pathway interaction strength between a given source cell and each of the seven other selected cell types as targets and are organized by source cell type: Endothelial ACKR1 (**a**), Differentiated PVL (**b**). Red bars indicate interactions in the >55 cohort; blue bars indicate interactions in the ≤55 cohort; text color indicates statistically significant enrichment via Wilcoxon rank-sum test (p-value < 0.05) using permutation-based CellChat communication probability calculations[30] in the >55 group (red), the ≤55 group (blue), or failure to achieve statistical significance (black). Y-axis depicts the scaled interaction probability.

Supplementary Figure 7

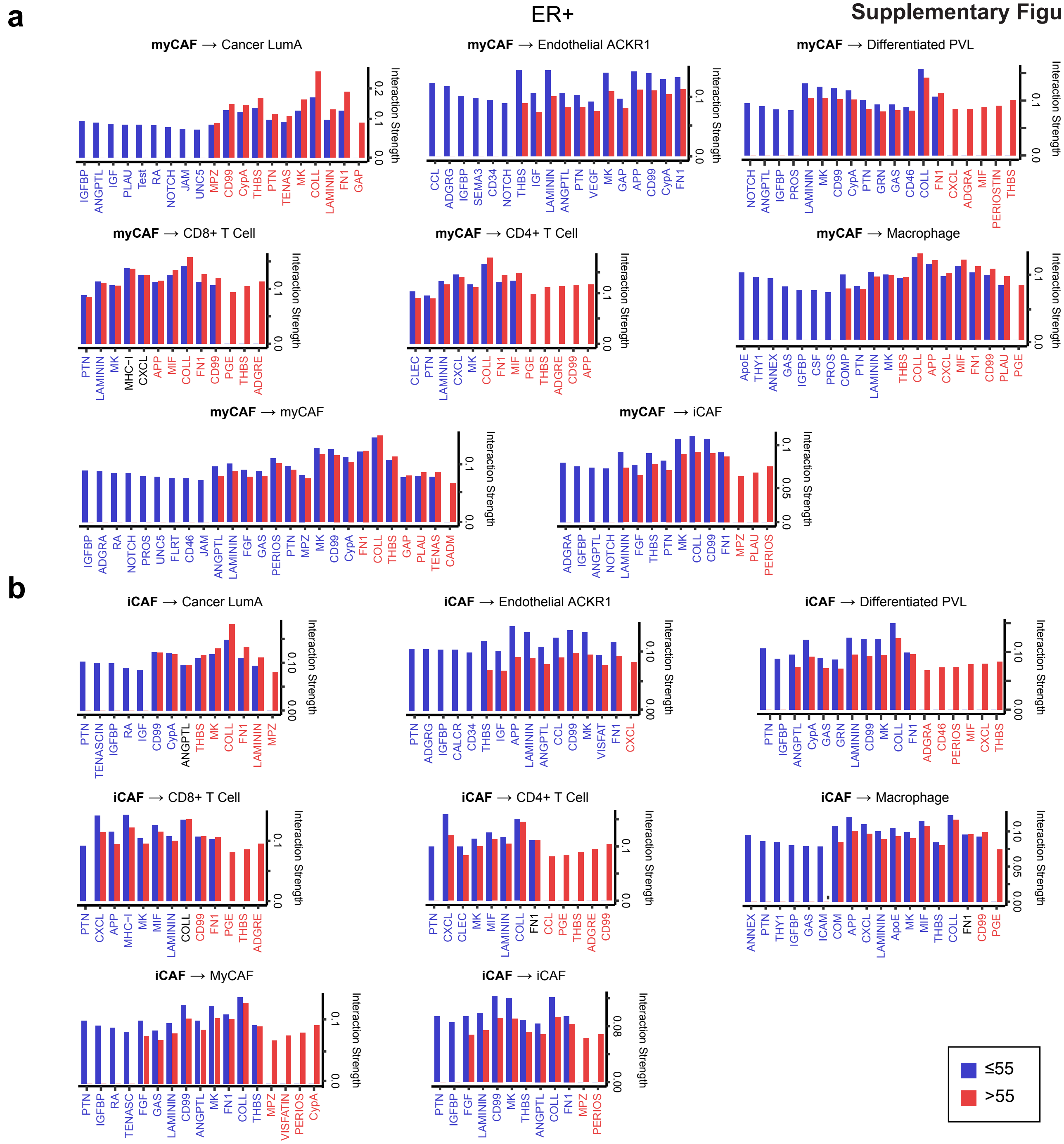

Supplementary Figure 7 | Age-related predicted signaling pathways between highly interactive cell types in ER+ Breast Cancer, CAFs (related to Fig. 6). Eight cell types with the highest differential interaction strengths were analyzed to identify specific biological interactions through which they were communicating using the CellChat *rankNet* function. **a-b**, Bar charts depict predicted signaling pathway interaction strength between a given source cell and each of the seven other selected cell types as targets and are organized by source cell type: myCAF (**a**), iCAF (**b**). Red bars indicate interactions in the >55 cohort; blue bars indicate interactions in the ≤55 cohort; text color indicates statistically significant enrichment via Wilcoxon rank-sum test (p-value < 0.05) using permutation-based CellChat communication probability calculations[30] in the >55 group (red), the ≤55 group (blue), or failure to achieve statistical significance (black). Y-axis depicts the scaled interaction probability.

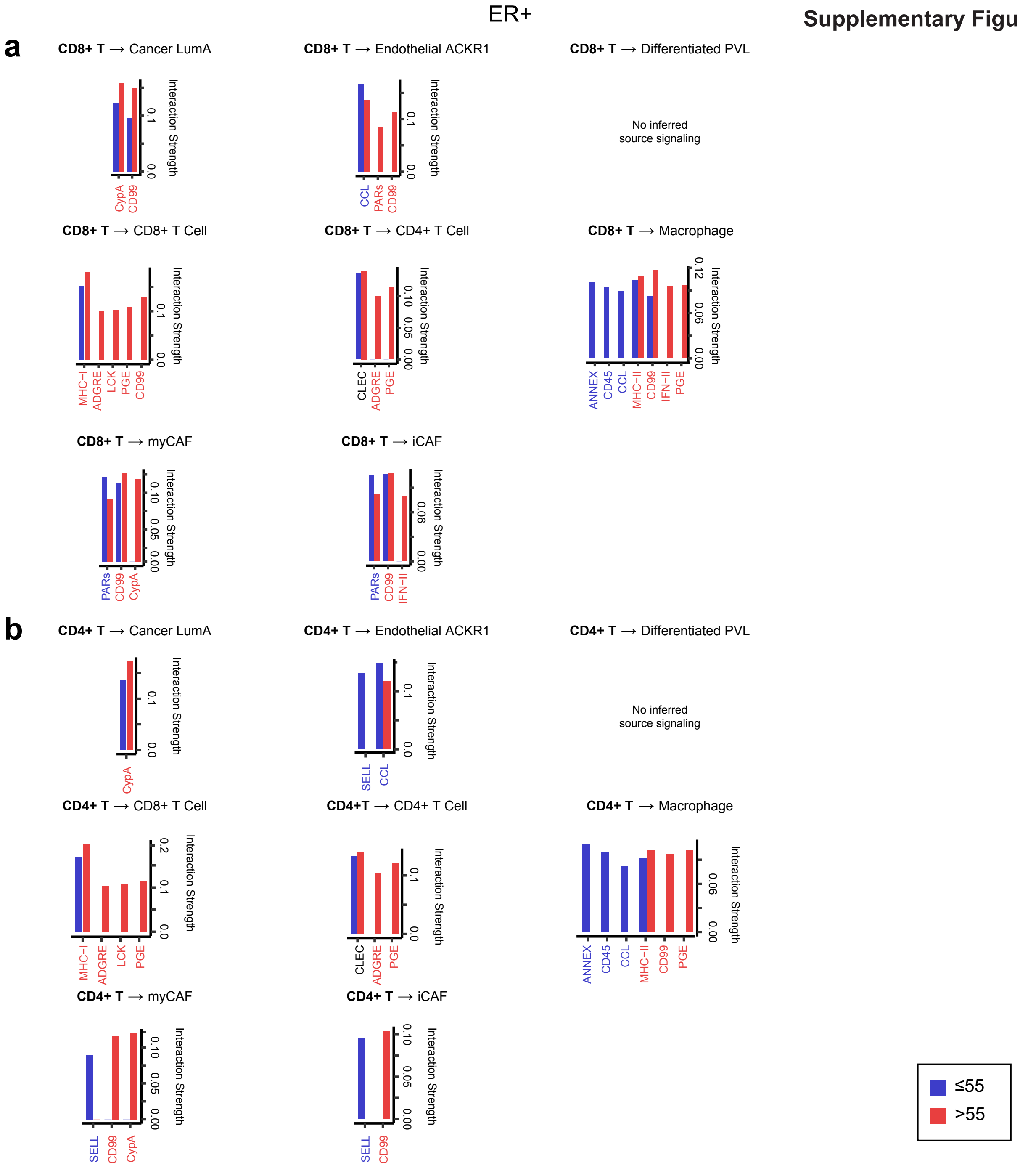

Supplementary Figure 8 | Age-related predicted signaling pathways between highly interactive cell types in ER+ Breast Cancer, T Cells (related to Fig. 6). Eight cell types with the highest differential interaction strengths were analyzed to identify specific biological interactions through which they were communicating using the CellChat *rankNet* function. **a-b**, Bar charts depict predicted signaling pathway interaction strength between a given source cell and each of the seven other selected cell types as targets and are organized by source cell type: CD8+ T Cells (**a**), CD4+ T Cells (**b**). Red bars indicate interactions in the >55 cohort; blue bars indicate interactions in the ≤55 cohort; text color indicates statistically significant enrichment via Wilcoxon rank-sum test (p-value < 0.05) using permutation-based CellChat communication probability calculations[30] in the >55 group (red), the ≤55 group (blue), or failure to achieve statistical significance (black). Y-axis depicts the scaled interaction probability.

ER+

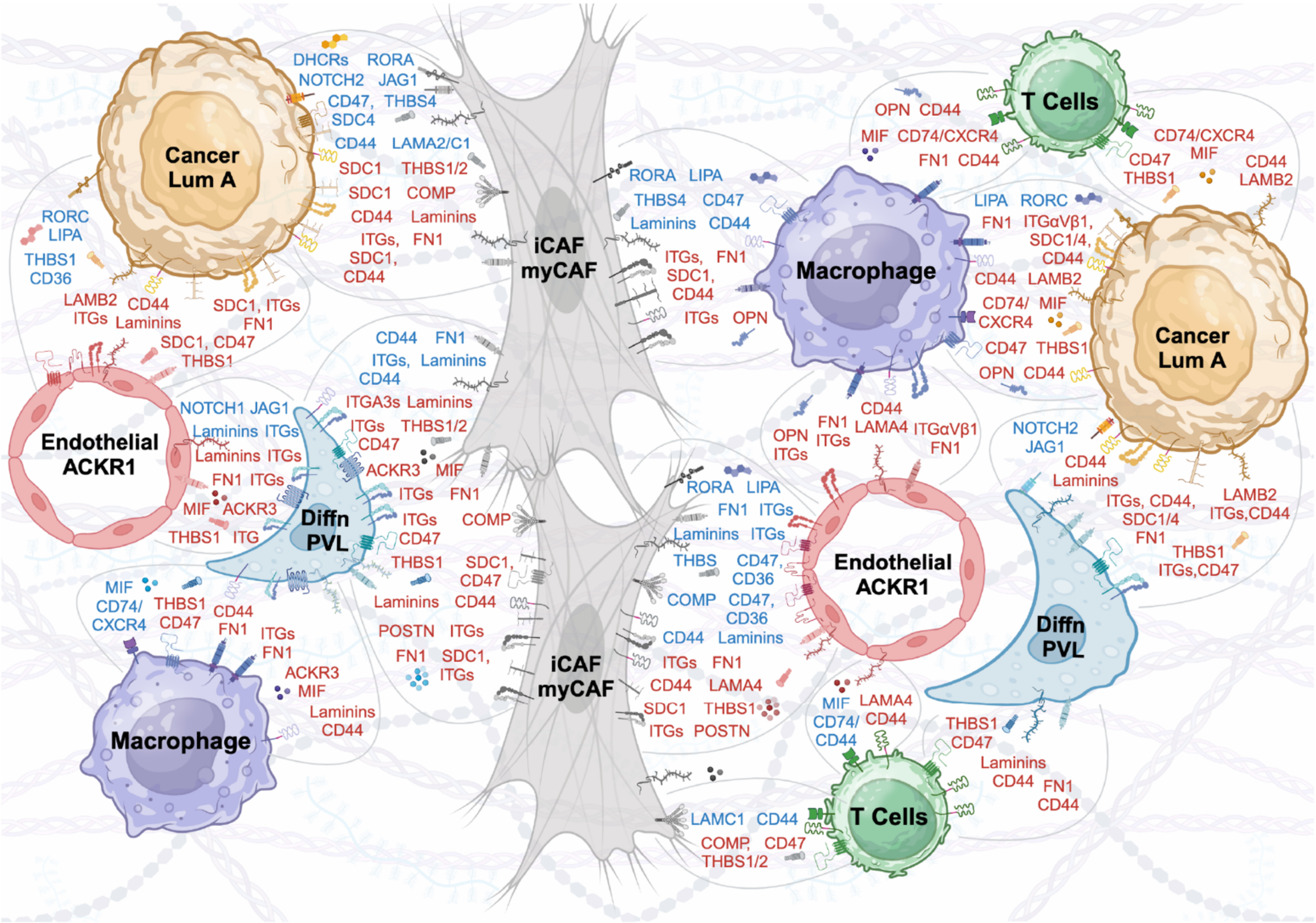

Supplementary Figure 9 | Age-associated signaling nodes in ER+ Breast Cancer. Schematic representation of the signaling nodes in ER+ Breast Cancer and additional signaling nodes of interest following manual curation of specific cell-cell interactions (See Methods, Source Data Table 5, and Supplementary Figs. 5-8). For clarity of representation, data were combined for iCAFs/myCAFs and CD8+/CD4+ T cells. Blue text indicates enrichment in the ≤55 age group; red text indicates enrichment in the >55 age group. Created in BioRender. McAllister, S. (2025) . https://BioRender.com/1xwgk2n.

Supplementary Figure 10

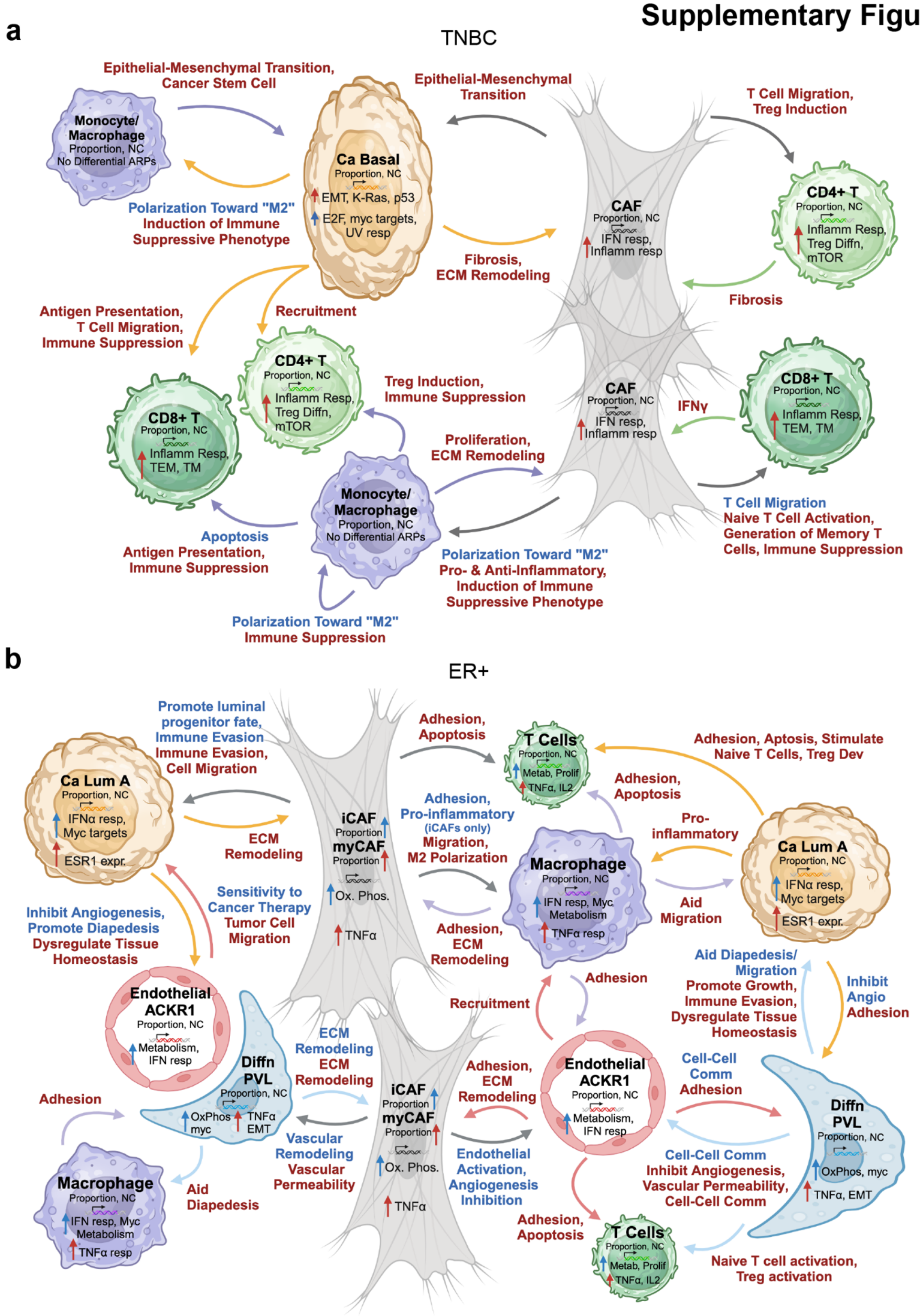

Supplementary Figure 10 | Working models of the age-related molecular landscapes of TNBC and ER+ breast cancer. a, b, Schematic depicts biologically distinct functions with age in the TNBC (**a**) and ER+ (**b**) breast tumor microenvironment. Selected cell types within the tumor microenvironment are shown with abundance, transcriptional (from METABRIC and ASPEN analyses), and communication (from CellChat analysis) differences with age. Arrows between cell types are colored to coincide with the source cell. Arrows within a cell type and all text depict enrichment in older (red) or younger (blue) patients. Created in BioRender. McAllister, S. (2025) https://BioRender.com/jynwnjb. McAllister, S. (2025) https://BioRender.com/mazax3z.
