# Supplementary material for: Cell populations in human breast cancers are molecularly and biologically distinct with age
Source: Nat Aging. 2025 Nov 4;5(12):2546–63. doi: 10.1038/s43587-025-00984-1 (PMC12705435; doi:10.1038/s43587-025-00984-1)
Supplement: Supplementary file 2 — Reporting Summary [file 43587_2025_984_MOESM2_ESM.pdf]

Corresponding author(s): Sandra McAllister, PhD and Peter van Galen, PhD

Last updated by author(s): Sep 9, 2025

Reporting Summary

Nature Portfolio wishes to improve the reproducibility of the work that we publish. This form provides structure for consistency and transparency in reporting. For further information on Nature Portfolio policies, see our Editorial Policies and the Editorial Policy Checklist.

Statistics

For all statistical analyses, confirm that the following items are present in the figure legend, table legend, main text, or Methods section.

n/a	Confirmed	
☐	☒	The exact sample size (*n*) for each experimental group/condition, given as a discrete number and unit of measurement
☐	☒	A statement on whether measurements were taken from distinct samples or whether the same sample was measured repeatedly
☐	☒	The statistical test(s) used AND whether they are one- or two-sided<br>*Only common tests should be described solely by name; describe more complex techniques in the Methods section.*
☐	☒	A description of all covariates tested
☐	☒	A description of any assumptions or corrections, such as tests of normality and adjustment for multiple comparisons
☐	☒	A full description of the statistical parameters including central tendency (e.g. means) or other basic estimates (e.g. regression coefficient) AND variation (e.g. standard deviation) or associated estimates of uncertainty (e.g. confidence intervals)
☐	☒	For null hypothesis testing, the test statistic (e.g. *F*, *t*, *r*) with confidence intervals, effect sizes, degrees of freedom and *P* value noted<br>*Give P values as exact values whenever suitable.*
☒	☐	For Bayesian analysis, information on the choice of priors and Markov chain Monte Carlo settings
☒	☐	For hierarchical and complex designs, identification of the appropriate level for tests and full reporting of outcomes
☐	☒	Estimates of effect sizes (e.g. Cohen's *d*, Pearson's *r*), indicating how they were calculated

Our web collection on statistics for biologists contains articles on many of the points above.

Software and code

Policy information about availability of computer code

Data collection	Image analysis was performed in Fiji/ImageJ v2.9.0 and QuPath v0.2.3.
Data analysis	All data analysis was conducted in R v4.4.1. Seurat v5 was used for CellChat analyses; all other Seurat analysis was completed in v4.4.0. CellChat v2.1.2. gage v2.54.0. fgsea v1.30.0. limma v3.60.6. ggplot2v 3.5.1 msigdbr v25.1.1 All analysis scripts are available at https://github.com/adrienneparsons/BC_singlecell_age. We also include a pseudocode document describing ASPEN in plain language in the same GitHub Repository. The folder “Reference Scripts” includes a more basic R script of the ASPEN framework to facilitate implementation for other single-cell RNA-seq datasets.

For manuscripts utilizing custom algorithms or software that are central to the research but not yet described in published literature, software must be made available to editors and reviewers. We strongly encourage code deposition in a community repository (e.g. GitHub). See the Nature Portfolio guidelines for submitting code & software for further information.

Data

Policy information about availability of data

All manuscripts must include a data availability statement. This statement should provide the following information, where applicable:

- Accession codes, unique identifiers, or web links for publicly available datasets
- A description of any restrictions on data availability
- For clinical datasets or third party data, please ensure that the statement adheres to our policy

All data necessary to interpret and verify the analyses in this study are available publicly through the original publication or as Source Data. The publicly available gene expression data for TNBC and ER+ breast cancer for METABRIC and Basal and Luminal A breast cancers in TCGA were accessed through cBioPortal 81–83. The TNBC and ER+ single-cell RNA sequencing data used in this study are publicly available and were accessed through GEO Accession number GSE176078 (https://www.ncbi.nlm.nih.gov/geo/query/acc.cgi?acc=GSE176078). Source data for figures have been provided in Excel format, citing related Figures in the file. The larger, gene expression files for METABRIC used in the analysis for Figure 1 can be found at https://figshare.com/articles/dataset/2024_METABRIC_TNBC_csv/27242253?file=4983452 and https://figshare.com/articles/dataset/2024_METABRIC_ER_csv/27242256?file=49834524
Remaining data supporting the findings of this study, including the mIF proximity data are part of a larger, unpublished clinical cohort. These data are available on reasonable request. All patients included provided written informed consent.

Research involving human participants, their data, or biological material

Policy information about studies with human participants or human data. See also policy information about sex, gender (identity/presentation), and sexual orientation and race, ethnicity and racism.

Reporting on sex and gender	Analyses were limited to females with breast cancer. Gender information was not available.
Reporting on race, ethnicity, or other socially relevant groupings	Not applicable
Population characteristics	Breast cancer patient age information is described in Source Data Tables 1, 3, and 4.
Recruitment	Not Applicable.
Ethics oversight	Tissue for the older cohorts (>70 years, grade I-III disease) were provided for secondary use in a de-identified manner under Mass General Brigham institutional review board approval 2021P001031, from the ELEVATE (clinical trial registration number: NCT03818087) and ADVANCE (clinical trial registration number: NCT03858322) studies. Original tissue collection was performed with institutional review board approval from all participating institutions and following the Declaration of Helsinki. All patients provided written informed consent. Tissue for the younger cohorts (<45 years, grade I-III) was commercially available from AMSBio. The ER+ TMA samples were from the Breast Boost cohort recruited to the St George Breast Boost study between 1998 and 2003 (Clinical Trials Registry NCT00138814). The TNBC TMA cohort consists of TNBC cases diagnosed between 2004 and 2019 at St George Hospital, Sydney, Australia. The TNBC cohort was not collected under a clinical trial. Ethics approval was granted by the South Eastern Sydney Local Health District Human Research Ethics Committee at the Prince of Wales Hospital, Sydney (Boost: HREC 96/16 and TNBC: HREC 2018/ETH00138) who granted a waiver of consent to perform research analyses on the tissue blocks. All methods were performed in accordance with the relevant institutional guidelines and regulations.

Note that full information on the approval of the study protocol must also be provided in the manuscript.

Field-specific reporting

Please select the one below that is the best fit for your research. If you are not sure, read the appropriate sections before making your selection.

☒ Life sciences ☐ Behavioural & social sciences ☐ Ecological, evolutionary & environmental sciences

For a reference copy of the document with all sections, see nature.com/documents/nr-reporting-summary-flat.pdf

Life sciences study design

All studies must disclose on these points even when the disclosure is negative.

Sample size	Sample sizes for computational methods relied on availability of publicly available datasets used in the analysis. Sample sizes for experimental validation relied on patient enrollment and sample availability. No statistical methods were used to determine sample sizes. The METABRIC cohorts consisted of n = 63 TNBC >65, n = 50 TNBC <45, n = 386 ER+ >65, and n = 86 ER+ <45. The TCGA cohort consisted of n = 37 basal >65, n = 30 basal < 45, n = 152 Luminal A >65, n = 68 Luminal A < 45. Cell Composition analyses, ASPEN, and CellChat analyses were performed on a cohort of n = 10 TNBC and n = 11 ER+ patients. Tissue mIF cohorts include n = 6 TNBC > 70, n = 5 TNBC < 45, n = 7 ER+ > 70, and n = 5 ER+ < 45. TMA analysis cohorts included n = 127 TNBC >55, n = 94 TNBC <=55, n = 237 ER+ > 55, n = 264 ER+ <=55.
Data exclusions	For computational analyses of the single-cell RNA seq atlas, we excluded HER2+ samples (n=5), because there were insufficient sample numbers and age ranges for ASPEN and downstream analyses. For bulk transcriptomic analyses of METABRIC and TCGA, we therefore also

excluded HER2+ patients, as well as patients with late stage or stage 0 disease. We also established prior to analysis to exclude METABRIC and TCGA patients between ages 45 and 65 years to align with established clinical risk. For TMAs, to account for potential misclassified cells, we excluded myCAFs, iCAFs, and endothelial cells identified within tumor regions, as well as tumor cells detected within stromal regions from the spatial analysis. TMA cores with <10% or >90% stromal region were excluded due to insufficient data, often associated with artifacts or missing morphology. In some ASPEN and CellChat analyses, exclusion criteria were pre-established, and some cell types were not analyzed due to insufficient representation of the cell type across the whole cohort, as described in the text.

Replication

All experiments and analyses were performed with biological replicates. Sample sizes are outlined in Source Data and within the text. All available data was analyzed, barring exclusions as described above. For mIF analyses, 5-9 images were analyzed per tumor. For TMA mIF, . each core was imaged once; mean cores analyzed for the ≤55 ER+ cohort: 2.5 (1-3 per patient); mean cores analyzed for >55 ER+ cohort: 2.27 (1-3 per patient); mean cores analyzed for the ≤55 TNBC cohort: 2.28 (1-3 per patient); mean cores analyzed for >55 TNBC cohort: 2.13 (1-3 per patient).

Randomization

Samples were analyzed based on established disease subtypes and age ranges, and all available tissue was analyzed per cohort, barring exclusions as described above. Therefore, randomization was not necessary for this study.

Blinding

Tissue-based assays and analyses were performed in a blinded fashion. For computational analysis of existing data, blinding was not performed, as it was necessary to classify samples into known subtype and age categories.

Reporting for specific materials, systems and methods

We require information from authors about some types of materials, experimental systems and methods used in many studies. Here, indicate whether each material, system or method listed is relevant to your study. If you are not sure if a list item applies to your research, read the appropriate section before selecting a response.

Materials & experimental systems

n/a	Involved in the study
☐	☒ Antibodies
☒	☐ Eukaryotic cell lines
☒	☐ Palaeontology and archaeology
☒	☐ Animals and other organisms
☒	☐ Clinical data
☒	☐ Dual use research of concern
☒	☐ Plants

Methods

n/a	Involved in the study
☒	☐ ChIP-seq
☒	☐ Flow cytometry
☒	☐ MRI-based neuroimaging

Antibodies

Antibodies used

For TMAs: panCK (1:2000, clone: AE1/AE3, host: Mouse, ab27988; Abcam), PDGFRβ (1:1000, CD140b, clone: Y92, host: Rabbit, ab32570; Abcam), αSMA (1:500, polyclonal, host: Rabbit, ab5694; Abcam), CD146 (1:1250, clone: EPR3208, host: Rabbit, ab75769; Abcam), THY1 (1:4000, CD90, clone: EPR3133, host: Rabbit, ab133350; Abcam), CD8 (1:1000, clone: C8/144B, host: Mouse, MA5-13473; Invitrogen), PD-1 (1:50, clone: EPR4877(2), host: Rabbit, ab137132; Abcam), and CD31 (1:100, clone: JC70A, host: Mouse, M0823; Agillent Technologies/DAKO). Lot numbers are unavailable; For additional mIF staining: Pan cytokeratin (1:100, clone: AE1/AE3, host: mouse, NBP2-2949; Lot 371P240806; Novus Biologicals), Vimentin (1:50, clone: SP20, host: rabbit, MA5-16409; Lot ZH4438378; Invitrogen), CD31 (1:50, clone: RM247, host: rabbit, MA5-33063; Lot ZL4575373; Invitrogen), COX4 (1:50, clone: 4D11-B3-E8, host: mouse, 11967S; Lot 4; Cell Signaling Technologies). Secondary antibodies: Donkey anti-Rabbit IgG (H+L) Highly Cross-Adsorbed Secondary Antibody, Alexa Fluor 488 (Invitrogen, A-21206, Lot 2330673), Goat anti-Mouse IgG (H+L) Highly Cross-Adsorbed Secondary Antibody, Alexa Fluor 594 (Invitrogen, A-21145, Lot 1736995), Goat anti-Mouse IgG (H+L) Highly Cross-Adsorbed Secondary Antibody, Alexa Fluor 647 (Invitrogen, A-21240, Lot 1772672), Goat anti-Rabbit IgG (H+L) Highly Cross-Adsorbed Secondary Antibody, Alexa Fluor 647 (Invitrogen, A-21244, Lot 2086678).

Validation

All primary antibodies were validated according to the manufacturer's associated product datasheet. For TMAs, Immunohistochemical (IHC) staining was used as a control to compare with mIF staining. Normal tissue cores were included as internal controls to ensure consistent staining intensity across all slides. During the multiplex optimization phase, antibody concentrations were further refined to standardize signal intensity where needed.

Plants

Seed stocks	Not applicable
Novel plant genotypes	Not applicable
Authentication	Not applicable
